# Heparin and Bivalirudin in Percutaneous Coronary Intervention for Acute Coronary Syndromes: A Review Article

**DOI:** 10.1155/cdr/5549914

**Published:** 2024-12-26

**Authors:** Guiping Wang, Kaijie Qi, Xuyang Li, Shuping Zuo, Ruolin Zhang, Yanan Zhao, Suya Sun, Juanjuan Zhang, Xiaokun Liu

**Affiliations:** ^1^Department of Cardiology, Tangshan Gongren Hospital, Tangshan, Hebei Province, China; ^2^School of Public Health, Wuhan University, Wuhan, Hubei Province, China; ^3^Harvard T.H. Chan School of Public Health, Harvard University, Boston, Massachusetts, USA

**Keywords:** ACS, bivalirudin, heparin, PCI

## Abstract

Acute coronary syndrome (ACS) is one of the most common leading global causes of mortality, encompassing ST-segment elevation myocardial infarction (STEMI), non-ST-segment elevation myocardial infarction (NSTEMI), and unstable angina (UA). Percutaneous coronary intervention (PCI) has become a pivotal therapeutic approach for ACS, underscoring the importance of anticoagulation strategies. Among the commonly employed anticoagulants in PCI, heparin and bivalirudin take precedence, with heparin serving as the archetypal choice. Nevertheless, the determination of an optimal anticoagulation regimen remains a point of contention in contemporary clinical practice. To address the differences in anticoagulants during PCI, we meticulously conducted a literature review through PubMed and Web of Science, employing search terms such as “heparin,” “bivalirudin,” “percutaneous coronary intervention,” and “acute coronary syndrome.” For patients with PIC brought on by STEMI, NSTEMI, and stable or UA pectoris, the review focused on randomized controlled trials to assess and compare the efficacy and safety of heparin and bivalirudin as anticoagulant options. This systematic review is aimed at furnishing valuable insights into the ongoing debate surrounding the choice of anticoagulation regimens in PCI. By scrutinizing clinical evidence derived from relevant trials, we seek to inform and guide healthcare practitioners in making informed decisions based on the unique requirements of patients with various ACS presentations.

## 1. Introduction

Acute coronary syndrome (ACS) is a major cause of death globally, encompassing disorders like ST-segment elevation myocardial infarction (STEMI), non-ST-segment elevation myocardial infarction (NSTEMI), and unstable angina (UA) [1]. The treatment of ACS with percutaneous coronary intervention (PCI) has become prevalent and successful [2]. Current guidelines emphasize the crucial role of appropriate anticoagulation therapy in averting thrombus formation and associated complications [3].

Two of the most popular anticoagulants used in PCI, heparin and bivalirudin, are categorized as Class I recommendations for PCI patients [4]. Common heparin is advantageous due to its low cost, ease of use, and straightforward management of complications, though it may increase the risk of bleeding. Moreover, certain patients cannot utilize heparin during PCI because of specific reasons, such as heparin resistance or allergies [5]. Bivalirudin, a synthetic small-molecule direct thrombin inhibitor, has gained widespread application in emergency medicine across Europe and the United States [6]. By directly and specifically inhibiting Factor IIa activity, it effectively prevents thrombosis, while carrying a potential risk of increasing the incidence of in-stent thrombosis [7]. Despite several advantages attributed to bivalirudin, including a short half-life that corresponds with a reduced risk of bleeding, linear pharmacokinetics, and minimal immunogenicity, its superiority over heparin remains a debate topic within the medical community [8].

Based on the most current clinical data and treatment results, this review is aimed at evaluating and contrasting the effectiveness and safety profiles of heparin and bivalirudin as anticoagulant agents in the context of PCI.

## 2. The Potential Mechanisms of Heparin and Bivalirudin in PCI

### 2.1. Mechanism of Action of Heparin

Heparin, an indirect thrombin inhibitor, primarily functions by binding to circulating antithrombin III, forming a complex that accelerates the inactivation of various coagulation factors, notably Factor IIa (thrombin) and Factor Xa, thereby manifesting its anticoagulant properties [9]. Due to its easy administration and quick commencement of action, heparin has traditionally been the anticoagulant of choice in PCI procedures. It is worth noting that heparin has also been associated with platelet activation, a consequence of its interaction with platelet membrane Glycoprotein (GP) IIb/IIIa receptors, which can precipitate heparin-induced thrombocytopenia, a significant adverse effect that necessitates careful monitoring [10].

### 2.2. Mechanism of Action of Bivalirudin

In order to prevent contact thrombosis and significantly extend the activation time of whole blood coagulation, bivalirudin, a direct thrombin inhibitor that is categorized as a hirudin derivative [11], directly and specifically inhibits Factor IIa activity [12]. In addition, bivalirudin is mainly metabolized by the kidneys and has a short half-life, making its effects reversible and short-lived. Its anticoagulant effect is easy to monitor and associated with a lower incidence of bleeding events, rendering it safer than conventional heparin anticoagulation [12]. The potential advantages of bivalirudin over heparin have garnered increased attention and focus. According to recent studies, bivalirudin dramatically lowers the incidence of bleeding events and possibly cardiac mortality while maintaining antithrombotic efficacy compared to heparin therapy in patients receiving direct PCI.

## 3. Heparin and Bivalirudin in PCI

### 3.1. Heparin and Bivalirudin in STEMI Patients

For patients with STEMI who need prompt reperfusion, primary percutaneous coronary intervention (PPCI) is the recommended treatment [13]. Unfractionated heparin (UFH) is recommended as a Class I antithrombotic agent by the European Society of Cardiology (ESC) and the American College of Cardiology Foundation/American Heart Association (ACCF/AHA) 2021 STEMI Guidelines, in the presence of heparin-induced thrombocytopenia with bivalirudin, or as a Class 2b agent in place of UFH to reduce bleeding [14]. Based on the Harmonizing Outcomes with Revascularization and Stents in Acute Myocardial Infarction (HORIZONS-AMI) trial, a multicenter study with 3602 patients, the recommendations highlight that bivalirudin monotherapy significantly reduced clinical adverse events (relative risk (RR) = 0.76, 95% confidence interval (CI) 0.63–0.92, *p* = 0.005) and major bleeding (RR = 0.6, 95% CI 0.46–0.77, *p* < 0.001) compared to UFH plus Glycoprotein IIb/IIIa inhibitor (GPI). However, no significant difference was found in 30-day stent thrombosis rates between the two groups (*p* = 0.3) [15].

The HORIZONS-AMI trial notably evaluated bivalirudin against the combination of UFH and GPI, while after the trial's publication, clinical practices shifted. Key factors influencing bleeding outcomes—such as reduced GPI usage, the standardized preference for potent P2Y12 inhibitors, and the increased adoption of radial over femoral access—have since been introduced into routine practice [16]. Therefore, while the application of the HORIZONS-AMI trial results in contemporary clinical practice may be limited, their validity remains unaffected. Based on this background, the European Ambulance Acute Coronary Syndrome Angiography (EUROMAX) study was conducted by Steg et al. Bivalirudin (loading dose of 0.75 mg/kg, maintenance dose of 1.75 mg/kg/h, reduced to 0.25 mg/kg/h after 4 h of maintenance) was compared with UFH in 2200 patients with STEMI treated with PCI, with the primary endpoint being death from any cause or noncoronary artery bypass grafting (non-CABG)–related hemorrhage within 30 days. The results showed that participants in the bivalirudin group had a higher risk of acute in-stent thrombosis but a lower frequency of mortality and serious clinical outcomes brought on by significant bleeding [17].

How Effective are Antithrombotic Therapies in Primary Percutaneous Coronary Intervention (HEAT-PPCI) trial, published in *The Lancet*, is a single-center, open, randomized study comparing bivalirudin and heparin in 1812 STEMI patients [18]. Patients were randomized 1:1 and stratified by age and cardiogenic shock presence. Bivalirudin was administered at 0.75 mg/kg loading and 1.75 mg/kg/h maintenance doses, while UFH was given at 70 U/kg. The primary efficacy outcome—a composite of all-cause mortality, cerebrovascular events, reinfarction, or unplanned revascularization—occurred in 8.7% of the bivalirudin group and 5.7% of the heparin group (absolute risk difference 3.0%; *RR* = 1.52; 95% CI 1.09–2.13; *p* = 0.01). The primary safety outcome, defined by Bleeding Academic Research Consortium criteria (Types 3–5), showed no significant difference, with major bleeding in 3.5% of the bivalirudin group versus 3.1% in the heparin group (RR = 1.15; 95% CI 0.70–1.89; *p* = 0.59). The findings suggest that heparin reduces major ischemic events in PPCI patients without increasing bleeding risk, offering potential cost savings over bivalirudin.

The managed invasively with and without ST elevation (MATRIX) trial, a large-scale, single-blind, multicenter RCT across Europe, included 7213 ACS patients (STEMI and NSTEMI). Patients were initially randomized to either transfemoral or transradial access PCI, followed by a second randomization to bivalirudin monotherapy or standard UFH with temporary GPIs [19]. Results showed no significant reduction in major adverse cardiovascular events (5.9% in the bivalirudin group vs. 6.5% in the heparin group; RR = 0.90, 95% CI 0.70–1.16, *p* = 0.43) or net adverse clinical events (7.0% vs. 8.2%; RR = 0.84, 95% CI 0.67–1.05, *p* = 0.13) among STEMI patients. Consequently, bivalirudin monotherapy did not lead to a significant reduction in the occurrence of major adverse cardiovascular events or net adverse clinical events among patients with STEMI. Nevertheless, bivalirudin demonstrated a significant reduction in bleeding rates. Specifically, bivalirudin significantly lowered bleeding rates, reducing Bleeding Academic Research Consortium (BARC) Type 3 and 5 bleeding in both STEMI (RR = 0.60, 95% CI 0.39–0.92, *p* = 0.019) and NSTEMI patients (RR = 0.47, 95% CI 0.26–0.85, *p* = 0.011) [20].

The efficacy of bivalirudin in reducing bleeding complications remains debated. The NAPLES III trial, a single-center, randomized, double-blind RCT, questioned bivalirudin's effectiveness in mitigating bleeding risk. In this study, 837 high-risk patients undergoing transfemoral PCI were randomized to either a heparin group (*n* = 419) or a bivalirudin group (*n* = 418). The primary outcome, major in-hospital bleeding, showed no significant difference between the two groups (RR = 0.78; 95% CI 0.35–1.72; *p* = 0.54), suggesting limited efficacy of bivalirudin in reducing bleeding [21]. The open-label VALIDATE-SWEDEHEART trial investigated the comparative effectiveness of bivalirudin versus heparin in 3005 STEMI patients undergoing PCI, with most procedures utilizing radial artery access. Patients were randomly allocated to either bivalirudin or heparin at the time of PCI, and 98% of participants also received a potent P2Y12 inhibitor. The primary endpoint—comprising all-cause mortality, myocardial infarction, or major hemorrhage—was assessed at 180 days. Results showed no significant difference in outcomes between the bivalirudin group (12.3%) and the heparin group (12.8%) (hazard ratio (HR) = 0.95; 95% CI 0.78–1.17; *p* = 0.64). These findings suggest that neither bivalirudin nor heparin demonstrated a clear advantage in reducing mortality or ischemic events [22]. Therefore, it remains uncertain whether bivalirudin or heparin is preferable for use during PCI in patients presenting with STEMI.

### 3.2. Heparin and Bivalirudin in NSTEMI Patients

A Swedish research team conducted an analysis using data from the SCAAR registry to compare 30-day mortality rates between bivalirudin and heparin in NSTEMI patients undergoing PCI. The study, encompassing 41,534 patients treated between 2005 and 2011, revealed that heparin was significantly associated with a reduction in 30-day mortality compared to bivalirudin [23]. These findings underscored the need for further validation through randomized controlled trials to substantiate the observed mortality benefit of heparin over bivalirudin.

The Acute Catheterization and Urgent Intervention Triage strategY (ACUITY) trial served as the first large-scale, prospective, randomized clinical study to evaluate anticoagulant strategies in patients with intermediate- to high-risk non-ST-elevation acute coronary syndrome (NSTE-ACS), enrolling 13,819 individuals, of whom 59% were diagnosed with NSTEMI. Participants were assigned to one of three treatment arms: UFH or enoxaparin with GPI, bivalirudin with GPI, and bivalirudin monotherapy. Results demonstrated no significant differences in the 30-day composite ischemic endpoint, major bleeding, or net clinical outcome between the heparin–GPI and bivalirudin–GPI groups. Notably, the bivalirudin monotherapy group showed a significant reduction in major bleeding rates without an associated increase in ischemic events, suggesting a potential safety advantage in terms of bleeding risk when compared with the heparin–GPI regimen [24].

Spontaneous Supratentorial Intracerebral Hemorrhage (SWITCH) III was a randomized, multicenter study designed to evaluate the safety of bivalirudin versus UFH in NSTEMI patients who were initially treated with sodium sulfadiazine heparan decarboxylate and urgent PCI. The results showed no significant differences in hemorrhagic and ischemic endpoints between the two groups. Bivalirudin proved to be noninferior to heparin in bleeding, death, myocardial infarction, acute revascularization, in-stent thrombosis, and reinfarction [25].

The Intracoronary Stenting and Antithrombotic Regimen: Rapid Early Action for Coronary Treatment 4 (ISAR-REACT 4) trial was a randomized controlled study designed to assess the noninferiority of bivalirudin compared to a combined regimen of heparin and abciximab in NSTEMI patients undergoing early invasive revascularization. Results indicated that combined antithrombotic therapy with heparin and abciximab did not significantly reduce the primary endpoint rate compared to bivalirudin monotherapy (RR = 0.96; 95% CI 0.74–1.25, *p* = 0.76). However, major bleeding was notably higher in the abciximab group, occurring in 4.6% of patients (40 patients) versus 2.6% in the bivalirudin group (22 patients) (RR = 1.84; 95% CI 1.10–3.07, *p* = 0.02). Bivalirudin demonstrated a favorable safety profile with significantly reduced rates of major and minor bleeding, while maintaining comparable anti-ischemic efficacy to the heparin–abciximab combination, with no elevated risk of stent thrombosis or mortality. Although the reduction in bleeding complications with bivalirudin did not correlate with improved 1-year survival rates, it still met noninferiority criteria relative to the combination therapy, offering comparable long-term outcomes, including mortality, myocardial infarction, and target vessel revascularization [26, 27].

The MATRIX trial results indicated no significant difference in major adverse cardiovascular events among NSTEMI patients treated with bivalirudin versus heparin. Specifically, 15.9% of patients in the bivalirudin group (253 patients) and 16.4% in the heparin group (262 patients) experienced these events (RR = 0.97; 95% CI 0.80–1.17; *p* = 0.74). Similarly, net adverse clinical events occurred in 16.5% of the bivalirudin group (262 patients) and 17.6% of the heparin group (281 patients), showing no significant difference between groups (RR = 0.93; 95% CI 0.77–1.12; *p* = 0.43) [20].

In summary, current studies indicate that, in PCI-treated NSTEMI patients, bivalirudin and heparin exhibit comparable efficacy, with no significant differences observed in bleeding incidence, mortality, myocardial infarction rates, or the occurrence of recurrent ischemia.

### 3.3. Heparin and Bivalirudin in Patients With UA

The HIRULOG trial, a double-blind, randomized study, evaluated the efficacy and safety of bivalirudin compared to heparin in 4098 patients undergoing angioplasty for unstable or postinfarction angina. Patients were assigned to receive either bivalirudin or heparin immediately prior to the procedure, with the primary endpoint defined as hospital mortality, myocardial infarction, vascular occlusion, or worsening cardiac function. While bivalirudin did not significantly reduce the rate of primary endpoint events relative to heparin, it was associated with a markedly lower incidence of bleeding complications (3.8% vs. 9.8%, *p* < 0.001). However, interpretation of these results is limited by the use of high-dose heparin and an older bivalirudin regimen, which may not reflect current interventional standards [28].

To evaluate the efficacy of the direct thrombin inhibitor bivalirudin relative to heparin in contemporary coronary interventions, the Complications with Hirulog for Ischemic Events Trial (CACHET) and Randomized Evaluation of PCI Linking Angiomax to Reduced Clinical Events-1 (REPLACE-1) trial were conducted. The CACHET demonstrated that bivalirudin, either alone or in combination with abciximab, was as safe and effective as low-dose heparin combined with abciximab [29]. In the REPLACE-1 trial, the composite efficacy endpoint—including death, myocardial infarction, or repeat revascularization within 48 h or before discharge—was observed in 5.6% of patients in the bivalirudin group and 6.9% in the heparin group (*p* = 0.40) [30]. This trail supports the safety and efficacy of bivalirudin, particularly when administered with planned GP IIb/IIIa blockade, thus validating this combination as a viable antithrombotic strategy in PCI.

The Randomized Evaluation of PCI Linking Angiomax to Reduced Clinical Events-2 (REPLACE-2) trial was conducted to extend the comparison of bivalirudin's efficacy against heparin combined with GPI over both 6-month and 12-month follow-up periods. Patients with both stable and UA as well as those with positive stress tests were recruited for this trial. Mortality at 12 months after enrollment and the incidence of death, myocardial infarction, or repeat revascularization at 6 months were the main outcomes. Results showed that the bivalirudin group's long-term clinical outcomes at 6 and 12 months were similar to those of the heparin group, confirming that bivalirudin is a useful substitute for PCI [31].

The purpose of the double-blind Intracoronary Stenting and Antithrombotic Regimen: Rapid Early Action for Coronary Treatment 3 (ISAR-REACT 3) trial was to evaluate the effectiveness of bivalirudin in comparison to heparin in patients who had previously received 325–500 mg of aspirin and 600 mg of clopidogrel. Four thousand five hundred seventy-one individuals with stable or UA receiving PCI were involved in the experiment. Death within 30 days, myocardial infarction, urgent revascularization of the target vessel because of myocardial ischemia, and significant in-hospital bleeding episodes were the main objectives. A decrease in these combined endpoints was considered the net clinical benefit. The incidence of mortality, myocardial infarction, or urgent revascularization of the target vessel were the secondary outcomes. Comparing bivalirudin to heparin at 30 days revealed no net clinical benefit or extra risk, indicating similar safety and effectiveness profiles for both medications in this context [32]. Information on randomized controlled trials in the text is summarized in Table 1.

In conclusion, while bivalirudin did not demonstrate a net clinical benefit in patients with stable or UA undergoing PCI after clopidogrel pretreatment, it significantly reduced the incidence of major bleeding. According to the latest ESC guidelines, bivalirudin lowers the risk of bleeding without impacting mortality and may thus be considered for administration in elderly patients with ACS, particularly those at elevated risk of hemorrhage [33, 34].

## 4. Conclusion

In PCI, the employment of a bivalirudin-based anticoagulation regimen demonstrated noninferiority to conventional heparin, with patients on bivalirudin showing a higher, though not statistically significant, incidence of myocardial infarction and in-stent thrombosis. However, a bivalirudin-based anticoagulation regimen significantly reduced the risk of bleeding in patients. Consequently, a bivalirudin-based anticoagulation regimen may be considered for patients at high risk of bleeding.

## Figures and Tables

**Table 1:** Study characteristic table.

**Study**	**Publication year**	**Country**	**Study details**	**Intervention (** **N** **)**	**Comparator (** **N** **)**	**Outcomes**
HORIZONS-AMI [15]	2008	US	RCT Total *N*: 3602 Median age: 60.2 Male: 76.6% STEMI: 100%	Bivalirudin: An IV bolus of 0.75 mg/kg, followed by an infusion of 1.75 mg/kg/h. No post-PCI continuation (*N* = 1800)	Heparin plus GPI: An IV bolus of 60 IU/kg, with subsequent boluses targeted to an activated clotting time of 200–250 s and abciximab or eptifibatide administered before PCI in all (*N* = 1802)	In patients with ST-segment elevation myocardial infarction who are undergoing primary PCI, anticoagulation with bivalirudin alone, as compared with heparin plus GPI, results in significantly reduced 30-day rates of major bleeding and net adverse clinical events
EUROMAX [17]	2013	European countries	RCT Total *N*: 2198 Median age: Bivalirudin: 61; control: 62 Male: Bivalirudin: 74.7%; control: 87.6%	Bivalirudin: An IV bolus of 0.75 mg/kg followed by an infusion of 1.75 mg/kg/h and continued for at least 4 h after PCI at 0.25 mg/kg/h (*N* = 1089)	Heparin: Either UFH (100 IU/kg without a GPI inhibitor or 60 IU/kg with a GPI) or an IV bolus of enoxaparin (*N* = 1109)	Bivalirudin, as compared with the control intervention, reduced the risk of the primary outcome (5.1% vs. 8.5%; RR, 0.60; 95% CI, 0.43–0.82; *p* = 0.001) and the principal secondary outcome (6.6% vs. 9.2%; RR, 0.72; 95% CI, 0.54–0.96; *p* = 0.02). Bivalirudin also reduced the risk of major bleeding (2.6% vs. 6.0%; RR, 0.43; 95% CI, 0.28–0.66; *p* < 0.001). The risk of acute stent thrombosis was higher with bivalirudin (1.1% vs. 0.2%; RR, 6.11; 95% CI, 1.37–27.24; *p* = 0.007). There was no significant difference in rates of death (2.9% vs. 3.1%) or reinfarction (1.7% vs. 0.9%)
HEAT-PPCI [18]	2014	United Kingdom	RCT Total *N*: 1812	Bivalirudin: A bolus of 0·75 mg/kg followed by an infusion of 1·75 mg/kg/h for the duration of the procedure. No post-PCI (*N* = 905)	Heparin: A bolus dose of 70 U/kg before the procedure. Additional doses if needed (*N* = 907)	Compared with bivalirudin, heparin reduces the incidence of major adverse ischemic events in the setting of PPCI, with no increase in bleeding complications
MATRIX [19]	2015	Italy, Netherlands, Spain, and Sweden	RCT Total *N*: 7213 STEMI: 55.6% NSTEMI: 44.4%	Bivalirudin 0.75 mg/kg bolus, 1.75 mg/kg/h infusion at the time of PCI (*N* = 3442) Duration: Terminated at the end of procedure	Heparin: 70–100 units/kg bolus in patients not receiving GPI or 50–70 units/kg bolus in patients receiving Glycoprotein IIb/IIIa inhibitors (*N* = 3473)	The rate of major adverse CV events was not significantly lower with bivalirudin than with heparin (10.3% and 10.9%, respectively; RR, 0.94; 95% CI, 0.81–1.09; *p* = 0.44), nor was the rate of net adverse clinical events (11.2% and 12.4%, respectively; RR, 0.89; 95% CI, 0.78–1.03; *p* = 0.12). Post-PCI bivalirudin infusion, as compared with no infusion, did not significantly decrease the rate of urgent target vessel revascularization, definite stent thrombosis, or net adverse clinical events (11.0% and 11.9%, respectively; relative risk, 0.91; 95% CI, 0.74–1.11; *p* = 0.34)
NAPLES III [21]	2015	Italy	RCT Single center Total *N*: 837	Bivalirudin 0.75 mg/kg bolus, 1.75 mg/kg/h infusion at the time of PCI (*N* = 418) Duration: Terminated at the end of procedure	UFH 70 units/kg bolus (*N* = 419)	There was no significant reduction in the incidence of major bleeding during the primary endpoint event primary period in the bivalirudin group compared with the plain heparin group (3.3% vs. 2.6%, *p* = 0.54) There was a lower probability of total bleeding events (8.1% vs. 9.1%, *p* = 0.71) in the bivalirudin group than in the plain heparin group, but the difference was not statistically significant There was no statistically significant difference in the probability of perioperative myocardial infarction between the bivalirudin and plain heparin groups (21.8% vs. 21.5%, *p* = 0.93)
VALIDATE-SWEDEHEART [22]	2021	Sweden	RCT Randomization followed a computer-generated list with 1:1 permuted block randomization stratified for STEMI/NSTEMI Total *N*: 3005 Average age: 66.8	Bivalirudin: Bolus of 0.75 mg/kg followed by an infusion of 1.75 mg/kg × h started as soon as possible and always before PCI of the culprit lesion (*N* = 1501)	Heparin: A total dose of 70–100 U/kg was recommended (*N* = 1504)	In patients with ST-segment elevation MI undergoing primary percutaneous coronary intervention with radial access and receiving current recommended treatments with potent P2Y12 inhibitors, the rate of the composite of all-cause death, MI, or major bleeding was not lower in those randomized to receive bivalirudin as compared with heparin
ACUITY [24]	2007	US	RCT Median time from admission to angiography = 20 h Total *N*: 13,819 Median age: 63 Male: 60% Race: NR 41% UA 59% NSTEMI 56% PCI 65% DES	Bivalirudin 0.1 mg/kg bolus, 0.25 mg/kg/h infusion at hospital admission (*N* = 4612) Duration: Terminated at the end of procedure	UFH 60 units/kg bolus, 12 units/kg/h infusion at hospital admission, goal ACT 200–250 s during PCI (48% of nonbivalirudin-treated patients received UFH) or enoxaparin 1 mg/kg SC twice daily at hospital admission, 0.3 mg/kg IV bolus if needed at the time of PCI (47% of nonbivalirudin-treated patients received LMWH) (*N* = 4603)	Substitution of unfractionated heparin or enoxaparin with bivalirudin results in comparable clinical outcomes in patients with moderate and high-risk acute coronary syndromes treated with GPI in whom percutaneous coronary intervention is done. Anticoagulation with bivalirudin alone suppresses adverse ischemic events to a similar extent as does heparin plus Glycoprotein IIb/IIIa inhibitors, while significantly lowering the risk of major hemorrhagic complications
SWITCH III [25]	2013	US and Canada	RCT Multiple sites Total *N*: 100 100% NSTEMI Average age: 62.7 ± 12.7 Male: 68%	Bivalirudin: Bolus of 0.75 mg/kg followed by an infusion of 1.75 mg/kg × h (*N* = 51)	UFH: Minimum dose of 60 U/kg IV for ACT > 200 s (> 200–250 s when Ilb/Illa administered) (*N* = 49)	Baseline clinical and angiographic characteristics were similar except for a higher body mass index in the UFH group (29.4 ± 4.7 vs. 27.3 ± 4.2, *p* = 0.02). Major bleeding was the primary outcome; a major bleeding event was documented in only 1 patient from the bivalirudin group (2%) and in none from the UFH group (*p* = 0.49). There was no death, Q-wave MI, or acute revascularization in either group
ISAR-REACT 3 [32]	2008	US, Europe	RCT Multiple sites Total *N*: 4571 Mean age: 67 Female: 23% Race: NR 18% UA 82% stable angina 88% DES, 5% BMS	Bivalirudin 0.75 mg/kg bolus, 1.75 mg/kg/h infusion at the time of PCI (*N* = 2289) Duration: Terminated at the end of procedure	UFH 100–140 units/kg bolus, placebo infusion at the time of PCI (*N* = 2281)	In patients with stable and UA who underwent PCI after pretreatment with clopidogrel, bivalirudin did not provide a net clinical benefit (it did not reduce the incidence of the composite endpoint of death, myocardial infarction, urgent target vessel revascularization, or major bleeding) as compared with unfractionated heparin, but it did significantly reduce the incidence of major bleeding
ISAR-REACT 4 [26]	2011	US, Europe	RCT 8 sites Randomized after initial angiography Total *N*: 1721 Mean age: 68 Female: 23% Race: NR 89% DES, 7% BMS 100% NSTEMI	Bivalirudin 0.75 mg/kg bolus, 1.75 mg/kg/h infusion at the time of PCI (*N* = 860) Duration: Terminated at the end of procedure	UFH 70 units/kg bolus Abciximab 0.25 mg/kg bolus, 0.125 mcg/kg/h infusion at the time of PCI Duration: Abciximab continued for 12 h post-PCI (*N* = 861)	Composite of death, large recurrent myocardial infarction, urgent target vessel revascularization, or major bleeding within 30 days after randomization
HIRULOG [28]	1995	North America and Europe	RCT 121 sites Total *N*: 4098 100% UA	Bivalirudin 1 mg/kg bolus, 4-h infusion at a rate of 2.5 mg/kg/h infusion and a 14–20-h infusion at a rate of 0.2 mg/kg/h infusion (*N* = 2059)	Heparin: 175 units/kg bolus, 15 units/kg/h (*N* = 2039)	Bivalirudin was at least as effective as high-dose heparin in preventing ischemic complications in patients who underwent angioplasty for UA, and it carried a lower risk of bleeding. Bivalirudin, as compared with heparin, reduced the risk of immediate ischemic complications in patients with postinfarction angina, but this difference was no longer apparent after 6 months
CACHET [29]	2002	US	RCT 2 sites Total *N*: 286 Stents were used in 88% of the patients. Abciximab was used in all the patients in the planned abciximab arms (heparin control and bivalirudin Phase A) and on a provisional basis in 34 of 144 patients (24%) in the bivalirudin arms of Phases B and C	Bivalirudin 0.75 mg/kg bolus, 1.75 mg/kg/h infusion at the time of PCI Phase A bivalirudin (1.0 mg/kg bolus, 2.5 mg/kg/h inf) + planned abciximab (*N* = 30) Phase B bivalirudin (0.5 mg/kg bolus, 1.75 mg/kg/h inf) + provisional abciximab (*N* = 85) Phase C bivalirudin (0.75 mg/kg bolus, 1.75 mg/kg/h inf) + provisional abciximab (*N* = 59)	Heparin: 70 units/kg bolus + planned abciximab (*N* = 94)	Bivalirudin with planned or provisional abciximab may be at least as safe and effective as low-dose heparin plus abciximab during percutaneous coronary intervention
REPLACE-1 [30]	2004	US	RCT 77 sites Total *N*: 1056 Male: Bivalirudin: 59.2%; heparin: 60.6% Caucasian: Bivalirudin: 88.2%; heparin: 91.5%	Bivalirudin 0.75 mg/kg bolus, 1.75 mg/kg/h infusion at the time of PCI (*N* = 532)	Heparin: Initial bolus of 60–70 U/kg, adjusted to achieve and maintain an activated clotting time of 200–300 s (*N* = 524)	The composite efficacy endpoint of death, myocardial infarction, or repeat revascularization before hospital discharge or within 48 h occurred in 5.6% and 6.9% of patients in the bivalirudin and heparin groups, respectively (*p* = 0.40). Major bleeding occurred in 2.1% vs. 2.7% of patients randomized to bivalirudin or heparin, respectively (*p* = 0.52)
REPLACE-2 [31]	2004	US, Canada, Europe	RCT 233 sites Total *N*: 1351 Mean age: 61 Female: 26% Race: 91% White 63% UA 89% BMS	Bivalirudin 0.75 mg/kg bolus, 1.75 mg/kg/h infusion at the time of PCI (*N* = 669) Duration: Terminated at the end of procedure	UFH 65 units/kg bolus GPI: Abciximab 0.25 mg/kg bolus, 0.125 mg/kg/h infusion Eptifibatide 180 mcg/kg double bolus, 2 mcg/kg/min infusion (*N* = 682)	Long-term clinical outcome with bivalirudin and provisional GP IIb/IIIa blockade is comparable with that of heparin plus planned GP IIb/IIIa inhibition during contemporary PCI

Abbreviations: ACE = angiotensin-converting enzyme; ACT = activated clotting time; BMS = bare metal stent; CV = cardiovascular; DES = drug-eluting stent; GP = glycoprotein; GPI = Glycoprotein IIb/IIIa inhibitor; h = hour/hours; kg = kilogram/kilograms; LMWH = low molecular weight heparin; mcg = microgram/micrograms; mg = milligram/milligrams; MI = myocardial infarction; min = minute/minutes; *N* = number of patients; NR = not reported; NSTEMI = non-ST-segment elevation myocardial infarction; PCI = percutaneous coronary intervention; RCT = randomized controlled trial; s = second/seconds; SC = subcutaneous; STEMI = ST-segment elevation myocardial infarction; U = unit/units; UA = unstable angina; UFH = unfractionated heparin; US = United States.

## Data Availability

Data availability is not applicable to this article as no new data were created or analyzed in this study.

## References

[1] Farag M., Gorog D. A., Prasad A., Srinivasan M. (2015). Bivalirudin versus unfractionated heparin: a meta-analysis of patients receiving percutaneous coronary intervention for acute coronary syndromes. *Open Heart*.

[2] Bhatt D. L., Lopes R. D., Harrington R. A. (2022). Diagnosis and treatment of acute coronary syndromes. *JAMA*.

[3] Bhogal S., Mukherjee D., Bagai J. (2020). Bivalirudin versus heparin during intervention in acute coronary syndrome: a systematic review of randomized trials. *Cardiovascular & Hematological Disorders Drug Targets*.

[4] O'gara P. T., Kushner F. G., Ascheim D. D. (2013). 2013 ACCF/AHA guideline for the management of ST-elevation myocardial infarction: a report of the American College of Cardiology Foundation/American Heart Association Task Force on Practice Guidelines. *Circulation*.

[5] Thygesen K., Alpert J. S., Jaffe A. S. (2012). Third universal definition of myocardial infarction. *Global Heart*.

[6] Erlinge D., Koul S., Omerovic E. (2019). Bivalirudin versus heparin monotherapy in non-ST-segment elevation myocardial infarction. *European Heart Journal Acute Cardiovascular Care*.

[7] Barria Perez A. E., Rao S. V., Jolly S. J. (2016). Meta-analysis of effects of bivalirudin versus heparin on myocardial ischemic and bleeding outcomes after percutaneous coronary intervention. *The American Journal of Cardiology*.

[8] Van Gameren M., Lemmert M. E., Wilschut J. M. (2018). An update on the use of anticoagulant therapy in ST-segment elevation myocardial infarction. *Expert Opinion on Pharmacotherapy*.

[9] Onishi A., Ange S. T., Dordick J. S., Linhardt R. J. (2016). Heparin and anticoagulation. *Frontiers in Bioscience-Landmark*.

[10] Zhang S., Gao W., Li H. (2016). Efficacy and safety of bivalirudin versus heparin in patients undergoing percutaneous coronary intervention: a meta-analysis of randomized controlled trials. *International Journal of Cardiology*.

[11] Shammas N. W. (2005). Bivalirudin: pharmacology and clinical applications. *Cardiovascular Drug Reviews*.

[12] Andreou C., Maniotis C., Koutouzis M. (2017). The rise and fall of anticoagulation with bivalirudin during percutaneous coronary interventions: a review article. *Cardiology and Therapy*.

[13] Steg P. G., James S. K., Atar D. (2012). ESC guidelines for the management of acute myocardial infarction in patients presenting with ST-segment elevation. *European Heart Journal*.

[14] Lawton J. S., Tamis-Holland J. E., Bangalore S. (2022). 2021 ACC/AHA/SCAI guideline for coronary artery revascularization: a report of the American College of Cardiology/American Heart Association Joint Committee on Clinical Practice Guidelines. *Circulation*.

[15] Stone G. W., Witzenbichler B., Guagliumi G. (2008). Bivalirudin during primary PCI in acute myocardial infarction. *The New England Journal of Medicine*.

[16] Kolh P., Windecker S., Alfonso F. (2014). 2014 ESC/EACTS guidelines on myocardial revascularization: the Task Force on Myocardial Revascularization of the European Society of Cardiology (ESC) and the European Association for Cardio-Thoracic Surgery (EACTS). Developed with the special contribution of the European Association of Percutaneous Cardiovascular Interventions (EAPCI). *European Journal of Cardio-Thoracic Surgery*.

[17] Steg P. G., Vant Hof A., Hamm C. W. (2013). Bivalirudin started during emergency transport for primary PCI. *The New England Journal of Medicine*.

[18] Shahzad A., Kemp I., Mars C. (2014). Unfractionated heparin versus bivalirudin in primary percutaneous coronary intervention (HEAT-PPCI): an open-label, single centre, randomised controlled trial. *Lancet*.

[19] Valgimigli M., Gagnor A., Calabró P. (2015). Radial versus femoral access in patients with acute coronary syndromes undergoing invasive management: a randomised multicentre trial. *Lancet*.

[20] Leonardi S., Frigoli E., Rothenbühler M. (2016). Bivalirudin or unfractionated heparin in patients with acute coronary syndromes managed invasively with and without ST elevation (MATRIX): randomised controlled trial. *BMJ*.

[21] Briguori C., Visconti G., Focaccio A. (2015). Novel approaches for preventing or limiting events (Naples) III trial: randomized comparison of bivalirudin versus unfractionated heparin in patients at increased risk of bleeding undergoing transfemoral elective coronary stenting. *JACC. Cardiovascular Interventions*.

[22] James S., Koul S., Andersson J. (2021). Bivalirudin versus heparin monotherapy in ST-segment-elevation myocardial infarction. *Circulation. Cardiovascular Interventions*.

[23] Angeras O., Koul S., Albertsson P. (2013). Heparin versus bivalirudin in patients with non ST-elevation acute coronary syndrome undergoing percutaneous coronary intervention - a report from SCAAR. *European Heart Journal*.

[24] Stone G. W., White H. D., Ohman E. M. (2007). Bivalirudin in patients with acute coronary syndromes undergoing percutaneous coronary intervention: a subgroup analysis from the Acute Catheterization and Urgent Intervention Triage strategy (ACUITY) trial. *Lancet*.

[25] Waksman R., Bertrand O., Driesman M. (2013). Bivalirudin versus unfractionated heparin during percutaneous coronary intervention in patients with non-ST-segment elevation acute coronary syndrome initially treated with fondaparinux: results from an international, multicenter, randomized pilot study (SWITCH III). *Journal of Interventional Cardiology*.

[26] Kastrati A., Neumann F. J., Schulz S. (2011). Abciximab and heparin versus bivalirudin for non-ST-elevation myocardial infarction. *The New England Journal of Medicine*.

[27] Schulz S., Kastrati A., Ferenc M. (2013). One-year outcomes with abciximab and unfractionated heparin versus bivalirudin during percutaneous coronary interventions in patients with non-ST-segment elevation myocardial infarction: updated results from the ISAR-REACT 4 trial. *EuroIntervention*.

[28] Bittl J. A., Strony J., Brinker J. A. (1995). Treatment with bivalirudin (Hirulog) as compared with heparin during coronary angioplasty for unstable or postinfarction angina. *The New England Journal of Medicine*.

[29] Lincoff A. M., Kleiman N. S., Kottke-Marchant K. (2002). Bivalirudin with planned or provisional abciximab versus low-dose heparin and abciximab during percutaneous coronary revascularization: results of the Comparison of Abciximab Complications with Hirulog for Ischemic Events Trial (CACHET). *American Heart Journal*.

[30] Lincoff A. M., Bittl J. A., Kleiman N. S. (2004). Comparison of bivalirudin versus heparin during percutaneous coronary intervention (the Randomized Evaluation of PCI Linking Angiomax to Reduced Clinical Events [REPLACE]-1 trial). *The American Journal of Cardiology*.

[31] Lincoff A. M., Kleiman N. S., Kereiakes D. J. (2004). Long-term efficacy of bivalirudin and provisional glycoprotein IIb/IIIa blockade vs heparin and planned glycoprotein IIb/IIIa blockade during percutaneous coronary revascularization: REPLACE-2 randomized trial. *Journal of the American Medical Association*.

[32] Kastrati A., Neumann F. J., Mehilli J. (2008). Bivalirudin versus unfractionated heparin during percutaneous coronary intervention. *The New England Journal of Medicine*.

[33] Byrne R. A., Rossello X., Coughlan J. J. (2023). 2023 ESC guidelines for the management of acute coronary syndromes. *European Heart Journal*.

[34] Melloni C., Jones W. S., Washam J. B. (2013). *AHRQ Comparative Effectiveness Reviews. Antiplatelet and Anticoagulant Treatments for Unstable Angina/Non–ST Elevation Myocardial Infarction*.

